# A prospective cohort study examining exposure to incarceration and cardiovascular disease (Justice-Involved Individuals Cardiovascular Disease Epidemiology – JUSTICE study): a protocol paper

**DOI:** 10.1186/s12889-022-12688-x

**Published:** 2022-02-16

**Authors:** Benjamin A. Howell, Lisa B. Puglisi, Jenerius Aminawung, Kirsten Bibbins- Domingo, Johanna Elumn, Colleen Gallagher, Nadine Horton, Dhruv S. Kazi, Harlan M. Krumholz, Hsiu-Ju Lin, Brita Roy, Emily A. Wang

**Affiliations:** 1 SEICHE Center for Health and Justice, Yale School of Medicine, New Haven, CT USA; 2 Section of General Internal Medicine, Yale School of Medicine, New Haven, CT USA; 3 Department of Epidemiology, University of California, San Francisco, California USA; 4 Connecticut Department of Correction, Wethersfield, CT USA; 5 Richard A. and Susan F. Center for Outcomes Research in Cardiology, Beth Israel Deaconess Medical Center, Boston, MA USA; 6 Harvard Medical School, Boston, MA USA; 7 Center for Outcomes Research and Evaluation, New Haven, CT USA; 8 Section of Cardiology, Department of Medicine, Yale School of Medicine, New Haven, CT USA; 9 Department of Social Work, University of Connecticut, Storrs, CT USA

**Keywords:** Incarceration, Prison, Cardiovascular Disease, Prospective Cohort Study

## Abstract

**Background:**

People who have been incarcerated have high rates of cardiovascular risk factors, such as hypertension and smoking, and cardiovascular disease (CVD) is a leading cause of hospitalizations and mortality in this population. Despite this, little is known regarding what pathways mediate the association between incarceration exposure and increased rates of CVD morbidity and especially what incarceration specific factors are associated with this risk. The objective of this study is to better understand CVD risk in people exposed to incarceration and the pathways by which accumulate cardiovascular risk over time.

**Methods and Analysis:**

The Justice-Involved Individuals Cardiovascular Disease Epidemiology (JUSTICE) study is a prospective cohort study of individuals released from incarceration with known cardiovascular risk factors. We are recruiting 500 individuals within three months after release from jail/prison. At baseline we are assessing traditional risk factors for CVD, including diet, exercise, and smoking, and exposure to incarceration-related policies, psychosocial stress, and self-efficacy. Cardiovascular risk factors are measured at baseline through point of care testing. We are following these individuals for the 12 months following the index release from incarceration with re-evaluation of psychosocial factors and clinical risk factors every 6 months. Using these data, we will estimate the direct and indirect latent effects of incarceration on cardiovascular risk factors and the paths via which these effects are mediated. We will also model the anticipated 10-year burden of CVD incidence, health care use, and mortality associated with incarceration.

**Discussion:**

Our study will identify factors associated with CVD risk factor control among people released from incarceration. Our measurement of incarceration-related exposures, psychosocial factors, and clinical measures of cardiovascular risk will allow for identification of unique targets for intervention to modify CVD risk in this vulnerable population.

**Supplementary Information:**

The online version contains supplementary material available at 10.1186/s12889-022-12688-x.

## Background

Cardiovascular disease (CVD) is the leading cause of death in the United States, though that risk is not spread evenly [1]. The presence of CVD or related risk factors, such as smoking and hypertension, is influenced by racialized social structures and policies, which affect most facets of life, including education, income, homeownership, employment, and access to healthcare [2]. One such structural underexplored determinant of the development and progression of CVD is incarceration.

**Fig. 1:**
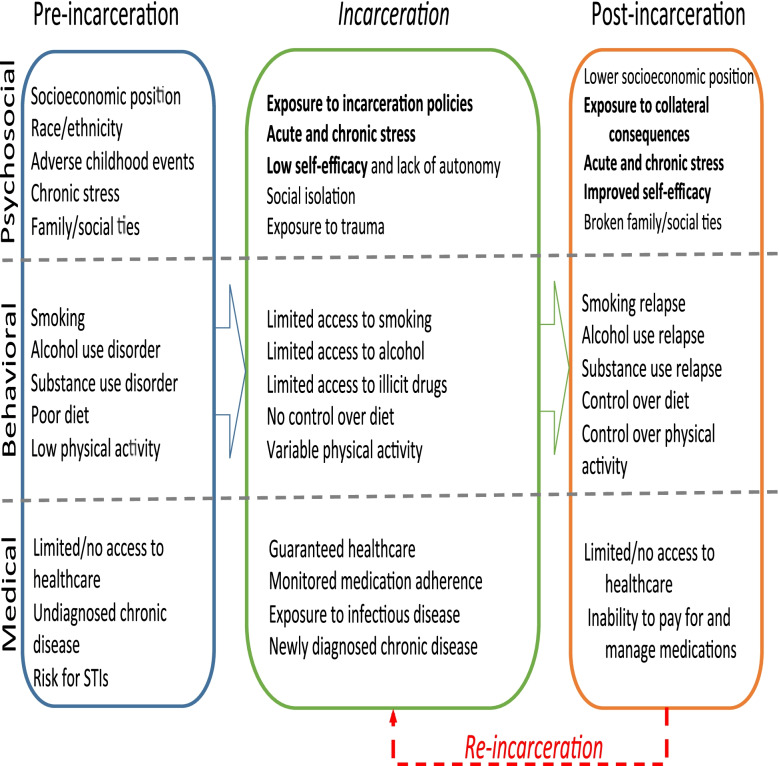
Psychosocial, Behavioral and Medical Risk factors for CVD risk factor control before, during and after the incarceration period; bolded text denotes population-specific psychosocial factors

The United States incarcerates more people per capita than any other country in the world [3] and at any given moment, over 2 million people are incarcerated in prisons and jails with another 5 million on parole or probation. While 2.7% of individuals living in the United States have a history of incarceration [4, 5], those from racial and ethnic minority groups are much more likely to be incarcerated [6], largely due to structural racism [7]. Studies have repeatedly shown that exposure to incarceration, ranging from being incarcerated [8–10], to having a family member incarcerated [11], and even living in a neighborhood with high rates of incarceration is associated with worse CVD outcomes. CVD is a major reason for hospitalization among people with a history of incarceration and it is a leading cause of death during incarceration and after release [12–14]. The factors (Fig. 1) that elevate CVD risk in this population are largely unknown and are only partly driven by a higher prevalence of conditions and risk factors associated with heart disease such as smoking [15, 16], diabetes, hypertension, and obesity [17, 18].

There is a dearth of factors in the literature that contribute to poor cardiovascular health for people with a history of incarceration, especially those that use direct measures of clinical risk factors. Findings from a prospective cohort study report that the experience of incarceration in young adulthood was associated with incident hypertension and increased risk of left ventricular hypertrophy, after controlling for sociodemographic, behavioral, and clinical risk factors [9]. A study in the Veterans Health Administration found that among veterans with a known history of hypertension, those who had been incarcerated in the past year were more likely to have poorly controlled hypertension [8]. Few national population-based studies or cohort studies evaluating CVD outcomes include questions on a history of incarceration, follow participants when they are incarcerated, or recruit among people incarcerated [19]. Therefore, there is a significant gap in knowledge about risk for cardiovascular outcomes in this population.

Exposure to the correctional environment and incarceration itself plausibly influences CVD risk. Incarceration, by design and in practice, is a stressful experience, given exposure to violence and solitary confinement [20], a general deprivation of freedom, and living in conditions that are often overcrowded and unsanitary. Incarceration fits into models that postulate a connection between chronic stress and allostatic load and CVD [21, 22]. Behavioral adaptations and consequences of living in stressful correctional environments, such as smoking or other drug use and sleep disturbances, may also play a role. Although access to health care is constitutionally mandated to those who are incarcerated [23], and may lead to earlier diagnosis of cardiovascular risk factors, long-standing and legitimate concerns exist about the quality and access of health care services available [24, 25]. In addition, health care in correctional settings, unlike community health care, is not primarily focused on prevention, patient education, or fostering patient self-efficacy [26].

The transition from correctional settings to the community leads to gaps in health care because of deficiencies in transitional services, lack of health care resources in communities to which individuals return, differential health insurance access [17], and the competing demands of reentry. For many, the period of incarceration can be followed by a period of community supervision (parole/probation), which involves continued surveillance and is also associated with poor health outcomes [27]. Even outside of periods of criminal supervision, people with a history of incarceration have differential access to educational opportunities, employment, housing, and healthy food [28] that is persistent and can affect chronic stress, self-efficacy in chronic disease management, and continued development of cardiovascular risk factors.

**Fig. 2:**
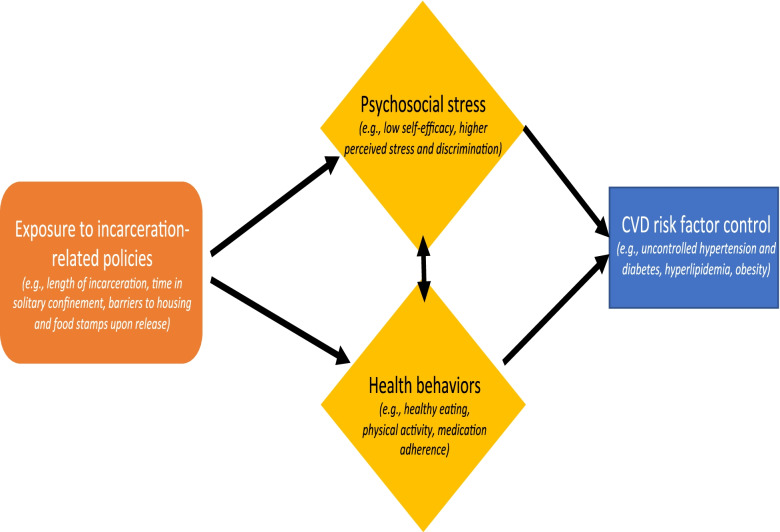
Hypothesized net effect of population-specific psychosocial factors, as well as behavioral risk factors on CVD risk factor control

The objective of this observational study is to better understand the impact of incarceration on CVD and the pathways by which people exposed to incarceration accumulate cardiovascular risk over time. Our study will examine the association between population-specific risk factors and CVD risk factor control (Fig. 2) in the immediate post-release period and examine how these factors evolve in the year following release. We will subsequently use the data collected to model the impact of these factors on 10-year and lifetime risk of CVD prevalence, hospitalizations, and mortality. We hypothesize that incarceration-related policies, perceived stress, and self-efficacy are associated with CVD risk factor control. We further hypothesize that exposure to longer periods of incarceration and stricter incarceration policies (such as solitary confinement) in tandem with the policies that limit access to social services after release result in increased chronic perceived stress, lower levels of self-efficacy, and subsequent poor cardiovascular risk factor control.

## Methods

### Study objectives

The primary objective of this study is to examine the association between population-specific risk factors and clinical evidence of control of CVD risk factors such as hypertension, hyperlipidemia, diabetes, and obesity. We are testing the hypothesis that high levels of stress, low self-efficacy, and exposure to certain criminal justice policies are associated with worse CVD risk factor control. Our secondary objective is to measure how changes in population-specific risk factors over time impact CVD risk factor control. We test the hypothesis that changes in population-specific factors mediate CVD risk factor control, independent of participant demographics and medical and behavioral risk factors. The third objective of the study is to estimate the impact of population-specific risk factors on long-term CVD morbidity and mortality. Using the Cardiovascular Disease Policy Model [29–31], an established simulation model of coronary heart disease and stroke incidence, prevalence, mortality, and costs in the US population, we test the hypothesis that change in population-specific risk factors, including psychosocial stress, self-efficacy, and exposure to certain criminal justice policies, augment future risk for CVD morbidity and mortality.

### Study design

This study is a prospective, observational cohort study. Participants are individuals with known CVD risk factors who have been recently released (within 30 days) from jail or prison.

### Setting

We recruit individuals released from jail or prison in New Haven, Bridgeport and Hartford, Connecticut. Connecticut has a unified prison and jail system and disproportionate incarceration of Black and Latinx individuals (70% of incarcerated population).

### Participants

Our goal is to recruit 500 individuals with known CVD risk factors after release from jail or prison to the community. We are working in partnership with the Connecticut Department of Correction (CT DOC) to pro-actively identify individuals being released from jail or prison who meet inclusion criteria (Table 1). The main criteria for inclusion into our study is presence of CVD or a modifiable risk factor, including hypertension, diabetes, obesity, or hyperlipidemia. The inclusion of only participants with baseline CVD risk factors both targets individuals at higher risk of poor health outcomes and improves the ability of our study to see an impact of various exposures, both during incarceration and in the community, on these risk factors.

**Table 1:** JUSTICE Study inclusion and exclusion criteria

Inclusion criteria:	Exclusion criteria:
Recently released from CT DOC (within three months)	Severe Mental Illness
At least one modifiable cardiovascular disease risk factor (diabetes, hyperlipidemia, hypertension, obesity) or cardiovascular disease	Terminal illness (anticipated death in < 1 year)
Returning to or residing in a study community (New Haven, Bridgeport, or Hartford, CT)	Intention to move out of study area in < 1 year

As cardiovascular risk factor control in the 12 months following release is a primary outcome, we do not include individuals with a known terminal illness and a life expectancy of less than 12 months. Finally, we exclude individuals with serious mental illness as they may be limited in their ability to consent to the study protocol. At the baseline visit, we obtain informed written consent. In order to verify understanding of study design and protocol, we use a teach-to-goal method which is developed for research participation among vulnerable populations [32].

### Follow-up & retention

Participants are followed monthly starting from month 2, with data collected at 3, 6, 9, and 12 months. Study contacts at 3 and 9 months are done via phone and study contact at 6 and 12 months are in-person, with modifications to include telephone or video visits as needed based on COVID-19 specific workplace adjustments. To maintain optimal contact with this hard-to-reach population, at months 2, 4, 5, 7, 8, 10, and 11 participants are contacted to update their contact information on file given significant “contact insecurity.” [33] For purposes of collecting data and contact for follow-up, at baseline, information including the individual’s name, aliases, home or cellular phone numbers, and addresses is collected. We collect date of birth, inmate number, social security number, and Medicaid ID number. In addition, we ask individuals to identify at least five people in their social network who could locate them, including at least one close friend or family member who does not live with them.

We contact participants 2 weeks prior to the in-person 6- and 12-month interviews with a follow-up telephone call or text. If after 3 telephone attempts, we are unable to contact the study participant we use other locators, reach out to support systems identified by participants at baseline, and use resources such as “reverse” telephone directories to contact participants for follow-up visits. Similar methods have been used with success (> 90% response rate) to follow individuals released from prison [34] or with active alcohol use disorder [35].

At baseline, participants are compensated $60 for the visit. During follow up, participants are credited $5 for each phone check-in, $20 for each phone interview, and $60 for each in-person interview. If participants are re-incarcerated during study follow-up, reimbursement for participation in study procedures are either sent to next of kin or held until after release to comply with CT DOC rules that do not permit research compensation while incarcerated.

### Baseline & follow-up data collection

Study visits at baseline, 6 and 12 months include data collection both via structured interviews and clinical data collection (including point of care testing). Data collected at baseline through the participant interviews includes socio-demographic data; clinical, psychosocial, and behavioral/medical factors; and exposure to incarceration policies. Clinical data collected include measurement of participant weight, height, and blood pressure. We perform point of care testing to objectively measure participant lipids, glycosylated hemoglobin, and urine toxicology. At 3 and 9 months follow up, participants are asked by phone about emergency department visits and overnight hospitalizations, and records are requested in cases of suspected CVD events.

In addition to information gathered from study participants directly, we also collect data from electronic health records and the CT DOC. We confirm emergency department visits and hospitalizations that occur during the follow-up period via electronic health records. Our research team also check publicly available data published by the CT DOC to assess if research participants have been re-incarcerated. If re-incarceration occurs, we contact the CT DOC to arrange a study visit (in person or mailed in survey) with the participant to complete 6- and 12-month study visits as needed. In the event of a participant’s death during study follow-up, we get consent from next of kin to obtain medical records and medical examiner reports to determine cause of death. We follow participant’s cardiovascular risk factor control and healthcare utilization through the electronic health records and healthcare utilization (for another 2 years or until the study’s end), after they have completed 12 months of follow up. This provides us additional data points which can be used to estimate long-term CVD morbidity and mortality.

We have included in Table 2 all the elements collected at baseline and follow-up. The baseline, 6- and 12-month follow-up survey instruments are included as Appendices.

**Table 2:** JUSTICE Study data collection elements measurements

Domain	Components/method of assessment	When Assessed	Purpose
**Demographic**	Age, sex, race, ethnicity, level of education	BL	Covariates
**Clinical**			
Blood pressure	Physical examination	BL, 6 and 12 months after release	Outcome
Height	Physical examination	BL	Outcome
Weight	Physical examination	BL, 6 and 12 months after release	Outcome
Lipid panel	Point of care blood test	BL, 6 and 12 months after release	Outcome
Glycosylated hemoglobin (HbA1c)	Point of care blood test	BL, 6 and 12 months after release	Outcome
**Psychosocial factors**			
Incarceration-related Correctional Policies	Exposure to solitary confinement, security level, co-payments for healthcare, civil-legal needs [36]	BL, and if re-incarcerated	Predictor
Incarceration-related Post-release policies	Self-reported barriers to housing, food stamp, licensure bans	BL, 6 and 12 months after release	Predictor
Self-efficacy	General Self Efficacy Scale [37]	BL, 6 and 12 months after release	Possible Mediator
Psychosocial stress	Perceived Stress Scale [38], Cumulative Adversity Interview [39]	BL, 6 and 12 months after release	Possible Mediator
Discrimination	Everyday Discrimination Scale [40]	BL, 6 and 12 months after release	Covariate
Autonomy and social support	Psychological Well-being scale [41], Personal wellbeing index [42], Cantril’s ladder [43]	BL, 6 and 12 months after release	Covariate
Post-traumatic stress disorder	PTSD symptom scale [44]	BL, 6 and 12 months after release	Covariate
Depression	Center for Epidemiologic Studies-Depression scale [45]	BL, 6 and 12 months after release	Covariate
Recidivism	Readmission into CT DOC	Weekly– from CT DOC	Covariate
**Behavioral factors**			
Physical activity	CARDIA self-report physical activity questionnaire [46]	BL, 6 and 12 months after release	Covariate
Diet	Eating at America's Table (EATS) "All Day Screener" [47]	BL, 6 and 12 months after release	Covariate
Smoking status	Lifetime smoking history, Current smoking history	BL, 6 and 12 months after release	Covariate
Substance use disorder	Addiction Severity Index, AUDIT, Rapid urine toxicology [48]	BL, 6 and 12 months after release	Covariate
**Medical factors**			
Primary Care Utilization	Medical records from CT DOC and Yale New Haven Health	BL, 6 and 12 months after release	Covariate
Medication Adherence	Pharmacy records from CT DOC/CMHC and Electronic Health Record, Morisky adherence [49]	BL, 6 and 12 months after release	Covariate

*BL* Baseline, *CT DOC* Connecticut Department of Corrections, *YNHH* Yale-New Haven Health, *CMHC* Connecticut Mental Health Center.

### Analytic approach

We will assess the associations between incarceration and cardiovascular risk factor control at baseline and associations over time between changes in population risk-factors and cardiovascular risk factor control. Using baseline data will evaluate cross-sectional associations between incarceration-related exposures, perceived stress, self-efficacy, and CVD risk factor control. We will examine bivariate associations between time incarcerated and exposure to certain incarceration policies (e.g., solitary confinement and others) and CVD related psychosocial factors (e.g., perceived stress, self-efficacy). Subsequently, we will examine bivariate associations between psychosocial factors and CVD risk factor control. Combining these results, we will undertake a path analysis using structural equation modelling to estimate the direct effects of incarceration exposures and indirect effects mediated through psychosocial factors. Our primary outcomes for this analysis will be any uncontrolled cardiovascular risk factor (SBP ≥ 140 or DBP ≥ 90, BMI ≥ 30, A1c ≥ 8, or LDL ≥ 160), and we will use logistic regression for these binary outcomes. Variance inflation factor (VIF) will be computed to measure the degree of multicollinearity among population-specific risk factors. If VIF indicates the existence of multicollinearity (i.e., VIF > 10 [50]), we will apply supervised machine learning models such as ridge or LASSO regression [51–53] to derive a more parsimonious model with a subset of predictors that are associated with CVD risk factor control.

To assess how changes in population-specific risk factors over time impact CVD risk factor control, we will perform 2-level hierarchical linear modeling using 12 months follow-up data to determine whether changes in factors specific to people who have been re-incarcerated are associated with worse CVD risk factor control. The 2-level hierarchical modeling approach will allow us to analyze data from both time-varying, repeated observations for each participant (level-1 data) and fixed characteristics for each participant (level-2 data). The time-varying exposures (perceived stress, self-efficacy, behavioral risk factors, recidivism) and time-fixed exposures (demographics, baseline incarceration exposures) will be analyzed together to estimate their impact on cardiovascular risk factors.

### Sample size

We estimated our sample with the following assumptions: 1) the ability to detect an odds ratio of 1.5 (equivalent to a small effect size), in uncontrolled pressure control (SBP ≥ 140 or DBP ≥ 90) between individuals with and without a population-specific risk factor; 2) two-sided 0.05 significance level; 3) an adjustment of R-squared of 0.4. Based on these assumptions, our study would need 308 participants to achieve 80% statistical power, or 413 to achieve 90% statistical power.

To estimate the number of participants needed for longitudinal 2-level hierarchical linear model analysis, we used an algorithm that takes into account the presence of correlated errors of measurement and person-specific effects, as well as the dropout rate, in estimating statistical power [54]. With a conservative assumption of a small to medium effect size, (d = 0.2), a significance level of 0.05, an intra-cluster correlation of 0.5, two random effects (intercept and slope for time effect), 80% power, three repeated measurements, a linear trend across time, and 0, 10, and 20% attrition rates, we would need 396, 420 and 456 participants at baseline respectively. Thus, a sample size of 500 would provide enough power to detect a small effect size with as much as 20% drop out rate.

We aim to oversample women in this study, as gender can be an important source of variation in population-specific factors. We will work with the only women’s correctional facility in Connecticut and reentry organizations for women to enroll more women. We will be able to conduct a stratified analysis for detecting a small effect size (d = 0.15), if we recruit at least 266 women (assuming 80% statistical power, two-sided test with significance level of 0.05).

#### Analytic approach: estimating cardiovascular disease morbidity and mortality

**Fig. 3:**
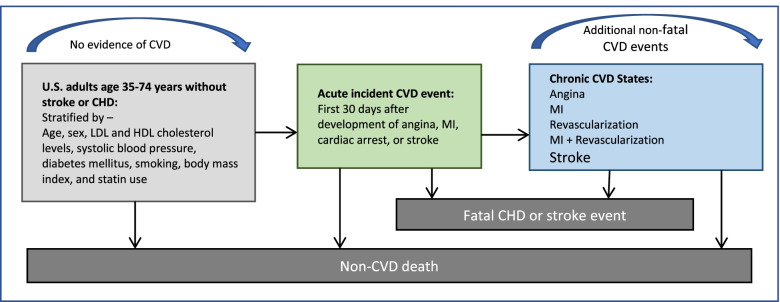
Schematic of the Cardiovascular Disease Policy Model. Abbreviations: CHD, coronary heart disease; CVD, cardiovascular disease; HDL, high-density lipoprotein; LDL, low-density lipoprotein; MI, myocardial infarction

We will model a 10-year projection of CVD events, CVD or non-CVD mortality, and health care costs as a function of age, sex, and clinical history, and estimate quality-adjusted survival, given the challenge of predicting trends further into the future. We will use all 12 months of data collected from the 500 participants and their health records and additional data on CVD risk factors control and healthcare utilization through the electronic health records. We will use a modified version the Cardiovascular Disease Policy Model, an established model of coronary heart disease and stroke outcomes (incidence, health care costs, mortality, Fig. 3) [29–31].

We will use the model to examine how exposure to incarceration-related policies, changes in psychosocial stress, and self-efficacy affect the risk of CVD morbidity and mortality. We will adopt a healthcare perspective and a 3% per year discount rate for future costs and outcomes. We will adhere to the recommendations of the Second Panel on Cost-Effectiveness in Health and Medicine [55]. Because of the younger age of the population included in this study, we will also model a lifetime analysis under varying assumptions of trends in risk factor prevalence and control. Our reference case will simulate the entire U.S. population of previously incarcerated individuals, but we will separately examine outcomes of interest among racial and ethnic minorities and whites in stratified analyses. We will also examine the effect of uncertainty in input parameters in deterministic and probabilistic sensitivity analyses.

The cost-effectiveness component of this model will take costs from a healthcare perspective and assume a 3% discount rate for future costs and outcomes [56]. Given the anticipated younger age of participants in the study, with CVD risk factors but likely lower rates of established CVD, we will model incidence of CVD based on estimates of risk in young adults based on cumulative exposure to cardiovascular risk factors from pooled cohorts. Our goal will be to model the CVD burden of the entire US population of previously incarcerated individuals, accounting for differences across racial and ethnic subgroups.

#### Validation of model assumptions

We will conduct analysis using linked administrative data from the CT DOC, CT Medicaid, and the National Death Index to describe the rates of CVD hospitalization and deaths to validate our CVD Policy Model estimates. We will include individuals who are Medicaid beneficiaries and have documented CVD risk factors in 2006, who were then incarcerated in CT DOC and subsequently released and follow them for 10 years to identify rates of morbidity and mortality using CT DOC data (morbidity while incarcerated), CT Medicaid (morbidity while in the community), and National Death Index. The administrative data will be used to establish the predictive and external validity of the CVD Policy Model. For the predictive validation, we will calculate the average simulated cumulative incidences of CVD events, CVD death, and non-CVD death for the 10-year period. For the external validation, we will determine the average simulated cumulative incidences of CVD events, CVD death, and non-CVD death for year 1 until year 10. We will recalibrate the centered cumulative baseline hazards and mean values of the risk factors from the CVD Policy Model to account for the potential differences with respect to the distribution of risk factors and CVD incidence between the CVD Policy Model and administrative data. Then we will compare the observed CVD event, CVD death, and non-CVD mortality incidences to determine if they match with the simulated incidences from the CVD Policy Model.

### Ethical considerations

All study procedures, materials, and protocols have been approved by the institutional review board of Yale University (HIC #2,000,022,213) and CT DOC Research Advisory Committee. Study data will be protected by a Certificate of Confidentiality. All efforts will be made by the research team, in collection, storage, analysis, and dissemination of data, to protect participant privacy and confidentiality. Our research team is unique in that we have a long experience including people with a history of incarceration in the design, implementation, conduct, analysis, and dissemination of our study findings.

### Data sharing

Consistent with NIH policy, we are planning to make the results of the study available to the research community and to the public at large. Final research data consisting of the computerized dataset, which do not contain any identifying personal health information, will be made available to other researchers on request and following acceptance for publication of the main findings from the final dataset per NIH guidelines. Documentation about the dataset, including information about the methodology and procedures used to collect the data, details about codes, definitions of variables, variable field locations, etc., will also be provided along with the final dataset.

### Dissemination plan

We plan to submit abstracts to present the research at annual scientific meetings in the fields of criminal justice and CVD epidemiology. Further, we plan to hold community meetings at local halfway houses and reentry organizations regarding study findings and prepare videos and pamphlets informing participants and the community. Finally, we will work with colleagues within the CT DOC and CT Medicaid to support current policies or encourage changes to policies to better support CVD risk factor management in this population.

## Anticipated results

If successful, our study will identify factors associated with poor CVD risk factor control in people released from incarceration. Our measurement of incarceration-related exposures, psychosocial factors, and clinical measures of cardiovascular risk will allow for identification of unique elements for intervention to modify CVD risk. In addition, following these individuals over the first year after release, we will be able to identify how these elements evolve over time, especially with respect to the impact of reincarceration. Finally, using our results, we will model 10-year cardiovascular outcomes and simulate the effect of interventions that impact incarceration exposures and psychosocial factors (self-efficacy, perceived stress). A better understanding of how cardiovascular risk is mediated in this population will both inform interventions to reduce this risk and potentially inform risk factor accumulation in other disenfranchised populations. This knowledge will also help inform potential population and individual-level interventions to reduce the preventable deaths due to CVD in this population.

## Conclusion

Despite the high prevalence of CVD and mortality in people with a history of incarceration, little is known about what factors related to incarceration exposure or subsequent mediating factors impact CVD progression. Whereas previous research has relied on self-report of CVD, our study will use measurement of incarceration exposure, psychosocial factors and directly-measured CVD risk factors at time of release from a correctional facility and in the subsequent year to measure the relationships between them. These findings will be used to better understand and model the impact of these factors on CVD burden in this population. These results will ultimately inform the development of interventions to improve CVD outcomes in people with a history of incarceration.

## Supplementary Information

**Additional file 1.** 

**Additional file 2.** 

## Data Availability

Consistent with NIH policy, we are planning to make the results of the study available to the research community and to the public at large. Final research data consisting of the computerized dataset, which do not contain any identifying personal health information, will be made available to other researchers on request and following acceptance for publication of the main findings from the final dataset per NIH guidelines. Documentation about the dataset, including information about the methodology and procedures used to collect the data, details about codes, definitions of variables, variable field locations, etc., will also be provided along with the final dataset.

## Abbreviations

A1c: Hemoglobin a1c
BMI: Body mass index
CHD: Coronary heart disease
CT DOC: Connecticut Department of Correction
CVD: Cardiovascular disease
DBP: Diastolic blood pressure
HDL: High-density lipoprotein
LASSO Regression: Least Absolute Shrinkage and Selection Operator regression
LDL: Low-density lipoprotein
SBP: Systolic blood pressure
VIF: Variance inflation factor
