# Supplementary material for: A prospective cohort study examining exposure to incarceration and cardiovascular disease (Justice-Involved Individuals Cardiovascular Disease Epidemiology – JUSTICE study): a protocol paper
Source: BMC Public Health. 2022 Feb 16;22:331. doi: 10.1186/s12889-022-12688-x (PMC8848673; doi:10.1186/s12889-022-12688-x)
Supplement: Supplementary file 1 — Additional file 1. [file 12889_2022_12688_MOESM1_ESM.docx]

# Cover page

Location: [ ] Bridgeport, CT [ ] Greenwich, CT [ ] New Haven, CT

| Initials of Interviewer: ___________ |
| --- |
| Administration Date: __________________ MM/DD/YYYY |

1. Participant referred from or by: [ ] Department of Correction

[ ] Re-entry program

[ ] Other; Specify

# Participant Name: Last _______________________ First

1. Date of birth? __________________ MM/DD/YYYY
2. Place of birth? City _____________________ State ___________

# Where do you live now? (If response is unclear, prompt to select category)

[ ] Homeless living on the street, park, bus station, etc.(living outdoors)

[ ] Homeless in a shelter

[ ] Drug treatment facility

[ ] Other residential facility or institution (e.g. mental health facility, halfway house)

[ ] Staying with family/friends

[ ] Rent an apartment/house

[ ] Own my home

[ ] Other (please specify) : ______________

1. What is your current address? (If homeless, include shelter name or common location where he/she sleeps)

Street: ________________ City/ Zip code: _____________________

1. What is your social security number? _________________
2. What is your inmate number? __________________
3. Do you have health insurance?

[ ]Yes  [ ] No [ ] Don’t know/ Not sure

9a. **If yes**, what kind of health insurance? (select all that apply)

[ ] Medicaid

[ ] Medicare

[ ] Employer-provided insurance [ ] VA Insurance

[ ] Other (please specify): ____________________

9b. **If Medicaid**, what is your Medicaid (*Husky* ) number? ________________________

9c. **If Medicare**, what is your Medicare number? __________________________

1. Is there telephone number through which we could reach you? Specifically, if we intend to contact you to remind you of an upcoming or missed appointment: Telephone number: _________________
2. Are there any other telephone numbers through which we could reach you? (friends, family, employer, parole/probation officer) *Please ask for 5 other contact numbers.*

Name: Name: Name: Name: Name:

Rel: Tel num: Rel: Tel num: Rel: Tel num: Rel: Tel num: Rel: Tel num:

# SECTION I: SOCIO-DEMOGRAPHICS

1. How do you describe yourself?

[ ] Male [ ] Female [ ] Transgender male

[ ] Transgender female [ ] prefer not to say [ ] prefer to self-describe ______________

1. How would you describe yourself? **(check all that apply)**

[ ] White [ ] African American/Black

[ ] Asian [ ] Native Hawaiian or other Pacific Islander

[ ] Native American or Alaskan Native [ ] Other (please specify): _________________

1. Do you consider yourself to be Hispanic or Latino/a?

[ ] Yes [ ] No

1. How would you describe your sexual orientation?

[ ] Straight/heterosexual [ ] Gay/lesbian/homosexual

[ ] Bisexual (attracted to both men and women) [ ] Asexual

[ ] Other (please specify): ___________ [ ] Prefer not to say

1. What is your current marital status?

[ ] Single/never married [ ] Married/living with a partner

[ ] Separated or Divorced [ ] Widowed

1. How much school did you complete?

[ ] 8^th^ grade or less [ ] Some high school

[ ] Graduate Equivalency Degree (GED) [ ] High school graduate

[ ] Some college or technical training [ ] College graduate

[ ] Graduate school

1. Are you currently receiving social security disability insurance?

*Note: Disabled: a person has a specific physical or mental condition that prevents the individual from working. The disability incapacitates a person and prevents him/her from doing any kind of work for at least 6 months.*

[ ] Yes [ ] No

1. Are you currently receiving food stamps (SNAP)?

[ ] Yes [ ] No [ ] Don’t know/Not sure

**(If Yes, or Don’t know, SKIP to question 20)**

19a. If No, what are the reasons you are not getting foods stamps?

[ ] Have not applied for food stamps [ ] Pending application

[ ] Criminal record (e.g. drug felony conviction) [ ] Don’t know

[ ] Other __________________________

1. Have you gone a whole day without food since your release from prison/jail because you did not have enough money to feed yourself?

[ ] Yes [ ] No **(SKIP to Q21)**

20a.**If Yes**, and receiving food stamps, what is the reason you went for a whole day without food?

[ ] Not enough to feed for a month [ ] Exchanged food stamps for alcohol/tobacco/drugs

[ ] Exchanged food stamps for other needs [ ] Other reason

# Last week, did you do any work for pay or profit? ( *Unpaid work in a family business is considered work.)*

[ ] Yes **(skip to Q22)**  [ ] No

[ ] Prefer not to answer **(skip to Q23)**

21a. If NO, What is the main reason you are not working? (Please select the response option that is closest to the patient’s answer/response) **(skip to Q23)**

[ ] Couldn’t find work/ Lacks necessary skills or experience

[ ] Discrimination due to criminal record

[ ] Other types of discrimination (includes age, race, sex or other)

[ ] Family responsibilities, including child care problems

[ ] In school or other training

[ ] Ill health or physical disability (only check if relates to OWN health/disability)

[ ] Has not started job search

1. How many hours per week do you USUALLY work at your job?

*Note: Usually is 50 percent of the time or more, or the most frequent schedule during the past 4 or 5 months. It is possible for someone who worked during the reference week to not usually work. If such cases, the usual hours is 0.*

[ ] Less than 8 hours [ ] 8 to 20 hours

[ ] 21- 34 hours [ ] 35 hours or more

[ ] Varies week to week

# What is your average monthly income *(include disability, food stamps, child support, workers comp, unemployment, any pensions, and money from family/friends)*?

[ ] No income [ ] $1-$99

[ ] $100-$499 [ ] $500-$999

[ ] $1000-$1999 [ ] $2000-$4999

[ ] $5000 or more [ ] Prefer not to say

# SECTION II: INCARCERATION HISTORY

1. Where did you serve your most recent incarceration? **(select all that apply)** [ ] Prison [ ] Jail
2. When did you start your last prison/jail term [the one that just ended]? ____________ (mm/dd/yyyy)
3. When were you released from your last prison/jail term? _____________ (mm/dd/yyyy)

# Are you currently on? (select all that apply)

[ ] Parole [ ] Probation [ ] Supervised Release

# If yes, when does the parole/probation/supervised release end? [If you are on both parole and probation, choose the furthest out date.] _____________ (mm/dd/yyyy)

1. During your last prison/jail term [the one that just ended], were you ever placed in restricted status (e.g. solitary, the hold, Seg, AdSeg, the SHU)?

[ ] Yes [ ] No [ ] Don’t know

**(If No, or Don’t know, SKIP to question 32)**

# What type(s) of restricted status were you on? [check all that apply]

[ ] Disciplinary action [ ] Protective Custody

[ ] Administrative Segregation (short-term) [ ] Special Risk Group (SRG)

[ ] Special Needs

# What is the total time that you spent on restricted status during your last prison/jail term?

[ ] 1 week or less [ ] 1-4 weeks

[ ] 1 to 3 months [ ] 3 to 6 months

[ ] 6 months-1 year [ ] More than 1 year

# When you were released from prison/jail, were you released directly from restricted status?

[ ] Yes [ ] No [ ] Don’t know

# Did you see a health care provider for a chronic health condition during your most recent prison/jail term?

[ ] Yes [ ] No **(skip to Q40)**

1. **If yes,** w*hen* I received care for my chronic conditions, I was: *(Patient Assessment of Chronic Illness Care (PACIC))*

|  |  | None of the time | A little of the time | Some of the time | Most of the time | Always |
| --- | --- | --- | --- | --- | --- | --- |
| A | Asked for my ideas when we made a treatment plan |  |  |  |  |  |
| B | Given choices about treatment to think about. |  |  |  |  |  |
| C | Asked to talk about any problems with my medicines or their effects. |  |  |  |  |  |
| D | Given a written list of things I should do to improve my health. |  |  |  |  |  |
| E | Satisfied that my care was well organized. |  |  |  |  |  |
| F | Shown how what I did to take care of myself influenced my condition. |  |  |  |  |  |
| G | Asked to talk about my goals in caring for my condition. |  |  |  |  |  |
| H | Helped to set specific goals to improve my eating or exercise. |  |  |  |  |  |
| I | Given a copy of my treatment plan |  |  |  |  |  |
| J | Encouraged to go to a specific group or class to help me cope with my chronic condition. |  |  |  |  |  |
| K | Asked questions, either directly or on a survey, about my health habits. |  |  |  |  |  |
| L | Sure that my doctor or nurse thought about my values, beliefs, and traditions when they recommended treatments to me. |  |  |  |  |  |
| M | Helped to make a treatment plan that I could carry out in my daily life. |  |  |  |  |  |
| N | Helped to plan ahead so I could take care of my condition even in hard times. |  |  |  |  |  |
| O | Asked how my chronic condition affects my life. |  |  |  |  |  |
| P | Contacted after a visit to see how things were going. |  |  |  |  |  |
| Q | Encouraged to attend programs in the correctional facility that could help me. |  |  |  |  |  |
| R | Referred to a dietitian, health educator, or counselor. |  |  |  |  |  |
| S | Told how my visits with other types of doctors, like an eye doctor or other specialist, helped my treatment. |  |  |  |  |  |
| T | Asked how my visits with other doctors were going. |  |  |  |  |  |

# Did you ever have to pay a fee (or co-pay) for medical care *(where you required to pay some amount of money when you saw a health care provider*)?

[ ] Yes [ ] No **(Skip to Q35)**

# Did you avoid seeking care because of this fee (or co-pay)?

[ ] Yes [ ] No [ ] Didn’t need care [ ] Don’t know

# Were you ever treated unfairly by healthcare providers (doctors, nurses, etc.)

# during your last prison/jail stay?

[ ] Yes [ ] No [ ] Not applicable

# Are there any medications that you were told to take regularly by the healthcare provider working with you during the last prison/jail term?

[ ] Yes [ ] No **(skip to Q40)** [ ] Refused **(skip to Q40)**

# Were you allowed to keep on you any of the medications that you were told to take regularly by the healthcare provider during the last prison/jail term?

[ ] Yes [ ] No

# Were you released from prison/jail with a medication prescription or voucher?

[ ] Yes [ ] No **(skip to Q40)**  [ ] Not sure **(skip to Q40)**

1. **If Yes**, approximately how many weeks of medication did you receive? _______ weeks

# Were you allowed to use any medical testing devices (such as blood pressure cuff or blood sugar meter) during the last prison/jail term?

[ ] Yes [ ] No **(skip to Q42)** [ ] Not needed/not applicable **(skip to Q42)**

# Were you allowed to keep any medical testing device on you (such as blood pressure or blood sugar meter) during the last prison/jail term?

[ ] Yes [ ] No [ ] Not applicable

1. Did you receive any education about caring for your disease condition [hypertension, diabetes, hyperlipidemia, obesity, CVD] during your most recent incarceration?

[ ] Yes [ ] No [ ] Don’t know

1. Did you work for pay during your most recent incarceration?

[ ] Yes [ ] No [ ] Prefer not to answer

1. Were you allowed exercise time during your most recent incarceration?

[ ] Yes [ ] No **(skip to question 48)**  [ ] Prefer not answer

1. How much time on average per week, were you allowed to exercise? ______ Hours ____Min
2. On average, how much time per week did you spend doing exercises that increase your heart rate, or make you sweat doing them, or make you breathe hard such as running, or playing basketball? ________ Hours _______Min
3. On average, how much time per week did you spend doing exercises that build or strengthen your muscles such as pushups or weight lifting? ________ Hours _______Min
4. During your most recent incarceration, how often did you eat the following:

|  | Never or < 1 per week | 1 -2 per week | 3 – 4 per week | 5 – 6 per week | 1 per day | 2 per day | 3 per day | 4 per day | 5+ per day |
| --- | --- | --- | --- | --- | --- | --- | --- | --- | --- |
| Foods cooked in fat (pan-fried, sautéed or deep fried) |  |  |  |  |  |  |  |  |  |
| A serving of vegetables? (don’t count salad, beans or potatoes) |  |  |  |  |  |  |  |  |  |
| A serving of fruit? (don’t count juice) |  |  |  |  |  |  |  |  |  |

1. How many times have you been incarcerated in your life (spent more than 1 night in jail/prison)

[ ] 1 time

[ ] 2 to 5 times

[ ] 6- 10 times

[ ] more than 10 times_______

1. How old were you the first time you were housed in a correctional facility (prison/jail)______ years.
2. How much time in your life have you spent in incarceration (including this past incarceration) ____years _____months?

# SECTION III: GENERAL HEALTH

# In general, would you say your health is:

# [ ] Excellent

# [ ] Very Good

# [ ] Good

# [ ] Fair

# [ ] Poor

# Has a doctor EVER told you that you have any of the following?

|  | Yes | No | Don't know | **Q53_1: If Yes,** were you diagnosed with this while in prison/jail | | | **Q53_2:If Yes,** at what age were you first told this? |
| --- | --- | --- | --- | --- | --- | --- | --- |
|  |  |  |  | Yes | No | Don't know | Age: years |
| a. Anemia or “low blood” |  |  |  |  |  |  |  |
| b. Angina or chest pain due to coronary heart disease |  |  |  |  |  |  |  |
| c. Heart attack or myocardial infarction |  |  |  |  |  |  |  |
| d. Congestive heart failure, also called weak heart or fluid on the lungs |  |  |  |  |  |  |  |
| e. Dementia or “Alzheimer’s” |  |  |  |  |  |  |  |
| f. Diabetes or high blood sugar |  |  |  |  |  |  |  |
| g. Liver disease or a bad liver or cirrhosis |  |  |  |  |  |  |  |
| h. Hepatitis C |  |  |  |  |  |  |  |
| i. HIV/AIDS |  |  |  |  |  |  |  |
| j. High cholesterol, lipids, or triglycerides |  |  |  |  |  |  |  |
| k. Hypertension or high blood pressure |  |  |  |  |  |  |  |
| l. Pancreatitis |  |  |  |  |  |  |  |
| m. Bad circulation in your legs or feet |  |  |  |  |  |  |  |
| n. Asthma |  |  |  |  |  |  |  |
| o. Chronic lung disease (emphysema, chronic bronchitis, or chronic obstructive lung disease) |  |  |  |  |  |  |  |
| p. Kidney failure (or bad kidneys) |  |  |  |  |  |  |  |
| q. Stroke or “mini” stroke (Transient Ischemic Attack) |  |  |  |  |  |  |  |
| r. Depression |  |  |  |  |  |  |  |
| s. Bipolar disorder |  |  |  |  |  |  |  |
| t. Post-traumatic stress disorder |  |  |  |  |  |  |  |
| u. Schizophrenia (hearing voices or seeing things others don’t) |  |  |  |  |  |  |  |
| v. Drug dependence/addiction |  |  |  |  |  |  |  |
| w. Alcohol dependence/addiction |  |  |  |  |  |  |  |
| x. Any kind of cancer (please specify): _ |  |  |  |  |  |  |  |
| z. Chronic pain |  |  |  |  |  |  |  |
| aa. Other (specify): _ _ |  |  |  |  |  |  |  |

1. Did you receive drug dependence medication assisted (naltrexone, suboxone, methadone) therapy while in prison/jail?

[ ] Yes

[ ] No

# Did you receive alcohol dependence medication assisted (naltrexone, suboxone, methadone) therapy while in prison/jail?

[ ] Yes

[ ] No

*Now we will turn to some questions about your mood and mental health.*

1. Over the last 2 weeks [or since release], how often have you been bothered by any of the following problems?

**[If patient responds not at all and/or several days to both questions a and b, skip to Q.57)**

| Over the last 2 weeks, how often have you been bothered by any of the following problems? | Not at all | Several days | More than half the days | Nearly everyday |
| --- | --- | --- | --- | --- |
| a. Little interest or pleasure in doing things |  |  |  |  |
| b. Feeling down, depressed, or hopeless |  |  |  |  |
| c. Trouble falling or staying asleep, or sleeping too much |  |  |  |  |
| d. Feeling tired or having little energy |  |  |  |  |
| e. Poor appetite or overeating |  |  |  |  |
| f. Feeling bad about yourself - or that you are a  failure or have let yourself or your family down |  |  |  |  |
| g. Trouble concentrating on things, such as reading the newspaper or watching television |  |  |  |  |
| h. Moving or speaking so slowly that other people might have noticed? Or the opposite - being so fidgety or restless that you have been moving around a lot more than usual |  |  |  |  |
| i. Thoughts that you would be better off dead or of hurting yourself in some way |  |  |  |  |

1. During your most recent incarceration, did you have any experience that was especially frightening, horrible, or upsetting (experience a traumatic event)?

[ ] Yes [ ] No [ ] Don’t know

**(If No, or Don’t know, SKIP to question 59)**

1. PTSD Symptom Scale (PSS)

Below is a list of problems that people sometimes have after experiencing a traumatic event. Please rate how much or how often these following things have occurred to you in the *last two weeks*

|  |  | Not at all | Once per week or less (a little bit) | 2-4 times per week (somewhat) | 5 or more times per week (very much) |
| --- | --- | --- | --- | --- | --- |
| A | Having upsetting thought or images about the traumatic event that come into your head when you did not want them to |  |  |  |  |
| B | Having bad dreams or nightmares about the traumatic event |  |  |  |  |
| C | Reliving the traumatic event (acting as if it were happening again) |  |  |  |  |
|  |  | Not at all | Once per week or less (a little bit) | 2-4 times per week (somewhat) | 5 or more times per week (very much) |
| D | Feeling emotionally upset when you are reminded of the traumatic event |  |  |  |  |
| E | Experiencing physical reactions when reminded of the traumatic event (sweating, increased heart rate) |  |  |  |  |
| F | Trying not to think or talk about the traumatic event |  |  |  |  |
| G | Trying to avoid activities or people that remind you of the traumatic event |  |  |  |  |
| H | Not being able to remember an important part of the traumatic event |  |  |  |  |
| I | Having much less interest or participating much less often in important activities |  |  |  |  |
| 7J | Feeling distant or cut off from the people around you |  |  |  |  |
| K | Feeling emotionally numb (unable to cry or have loving feelings) |  |  |  |  |
| L | Feeling as if your future hopes or plans will not come true |  |  |  |  |
| M | Having trouble falling or staying asleep |  |  |  |  |
| N | Feeling irritable or having fits of anger |  |  |  |  |
| O | Having trouble concentrating |  |  |  |  |
| P | Being overly alert |  |  |  |  |
| Q | Being jumpy or easily startled |  |  |  |  |

# Have these problems interfered with any of the following?

# *(if any of the above items scored more than “Not at all”)*

|  | No | Yes |
| --- | --- | --- |
| Work |  |  |
| Household duties |  |  |
| Friendships |  |  |
| Fun/leisure activities |  |  |
| Schoolwork |  |  |
| Family Relationships |  |  |
| Sex life |  |  |
| General life satisfaction |  |  |
| Overall functioning |  |  |

*Now we will turn to questions on healthcare utilization*

SECTION IV: HEALTH CARE UTILIZATION

# Before your last incarceration, did you have a usual source of medical care? (A place you go if you need check-up or if you are ill)

[ ] Yes

[ ] No, I don’t seek routine care anywhere **(skip to 62)**

[ ] No, I seek routine care at more than one place **(skip to 62)**

# If yes, how would you describe the kind of place where you sought routine or non-emergent care? (check only one)

[ ] Doctor’s office [ ] Clinic or health center

[ ] Emergency room [ ] Some other place, Specify ______________

# Did you have health insurance right before going to last prison/jail stay?

[ ] Yes [ ] No **(Skip to 64)**  [ ] Don’t know **(Skip to 64)**

# What type of health insurance? (Check all that apply).

[ ] Medicaid or Husky (CT) [ ] Medicare

[ ] Employer provided health insurance [ ] VA insurance

[ ] Other (please specify): ______ [ ] Don’t know

# Do you have a primary care doctor (A doctor you go to regularly if you need check-up or are ill)?

[ ] Yes [ ] No [ ] Don’t know /Not sure

1. Have you seen a primary care doctor/provider since release from prison/jail?

[ ] Yes [ ] No **(skip to Q67)**

1. If yes, w*hen* I received care for my chronic conditions, I was: *(Patient Assessment of Chronic Illness Care (PACIC))*

|  |  | None of the time | A little of the time | Some of the time | Most of the time | Always |
| --- | --- | --- | --- | --- | --- | --- |
| A | Asked for my ideas when we made a treatment plan |  |  |  |  |  |
| B | Given choices about treatment to think about. |  |  |  |  |  |
| C | Asked to talk about any problems with my medicines or their effects. |  |  |  |  |  |
| D | Given a written list of things I should do to improve my health. |  |  |  |  |  |
| E | Satisfied that my care was well organized. |  |  |  |  |  |
| F | Shown how what I did to take care of myself influenced my condition. |  |  |  |  |  |
| G | Asked to talk about my goals in caring for my condition. |  |  |  |  |  |
| H | Helped to set specific goals to improve my eating or exercise. |  |  |  |  |  |
| I | Given a copy of my treatment plan |  |  |  |  |  |
| J | Encouraged to go to a specific group or class to help me cope with my chronic condition. |  |  |  |  |  |
| K | Asked questions, either directly or on a survey, about my health habits. |  |  |  |  |  |
| L | Sure that my doctor or nurse thought about my values, beliefs, and traditions when they recommended treatments to me. |  |  |  |  |  |
| M | Helped to make a treatment plan that I could carry out in my daily life. |  |  |  |  |  |
| N | Helped to plan ahead so I could take care of my condition even in hard times. |  |  |  |  |  |
|  |  | None of the time | A little of the time | Some of the time | Most of the time | Always |
| O | Asked how my chronic condition affects my life. |  |  |  |  |  |
| P | Contacted after a visit to see how things were going. |  |  |  |  |  |
| Q | Encouraged to attend programs in the community that could help me. |  |  |  |  |  |
| R | Referred to a dietitian, health educator, or counselor. |  |  |  |  |  |
| S | Told how my visits with other types of doctors, like an eye doctor or other specialist, helped my treatment. |  |  |  |  |  |
| T | Asked how my visits with other doctors were going. |  |  |  |  |  |

1. Are you currently taking any medications that you were told to take regularly by a healthcare provider?

[ ] Yes [ ] No [ ] Refused

**(If No, or Refused, SKIP to question 69)**

1. Abbreviated (4 item) Morisky scale

|  | Yes | No |
| --- | --- | --- |
| a. Do you ever forget to take your medications? |  |  |
| b. Are you careless at times about taking your medications? |  |  |
| c. When you feel better do you stop taking your medications? |  |  |
| d. Sometimes if you feel worse when you take your medication, do you stop taking it? |  |  |

# Have you been to an emergency department since being released from prison/jail?

[ ] Yes [ ] No **(skip to Q71)**

# How many times (ED since release)?

1. Have you been hospitalized (24 hours/admitted overnight) since being released from prison/jail?

[ ] Yes [ ] No

#

*Now we will turn to a few questions about smoking, alcohol use, illicit drug use, and your activities following release from prison/jail.*

SECTION V: SUBSTANCE USE AND RISK BEHAVIORS

# Tobacco Use

1. Have you ever smoked cigarettes?

[ ] Yes [ ] Never smoked **(skip to 78)**

1. Have you smoked at least 100 cigarettes in your entire life?

[ ] Yes [ ] No [ ] Don’t know/Not sure

# Did you smoke cigarettes regularly before your last incarceration? By “regularly”, we mean at least 5 cigarettes per week, almost every week.

[ ] Yes [ ] No **(skip to 75)**

# Did you receive nicotine replacement therapy (nicotine patches, nicotine gum, etc.) while in prison/jail?

[ ] Yes [ ] No

# Do you currently smoke cigarettes?

[ ] Yes [ ] No **(skip to 77)**

# On average, about how many cigarettes a day do you now smoke? cigarettes

# Alcohol Use

# Please answer the following questions about your alcohol use since release (AUDIT-C)

|  | Question | Response Options | | | | |
| --- | --- | --- | --- | --- | --- | --- |
| A | How often do you have a drink containing alcohol? | Never  □  \\ | rarely (<1/week) (monthly or less)  □ | about once a week (2-4x/mo)  □ | 2 to 3 times a  Week  □ | 4 or more times a week  □ |
| B | How many standard drinks containing alcohol do you have on a typical day? | 1 or 2  □ | 3 or 4  □ | 5 or 6  □ | 7 to 9  □ | 10 or more  □ |
| C | How often do you have six or more drinks on one occasion? | Never  □ | less than monthly  □ | Monthly  □ | Weekly  □ | daily or almost daily  □ |

# Illicit Drug Use

1. For each of the following drugs, indicated use since release and lifetime use of the drugs and the usual route.

|  |  |  |  | Note the usual or most recent route. *For more than one route, choose the most severe. The routes are listed from least severe (left) to most severe (right)* | | | | |
| --- | --- | --- | --- | --- | --- | --- | --- | --- |
|  | Name of Drug | Use since release (days) | Lifetime use (years) | Oral | Nasal | Smoking | Non-IV injection | IV |
| A | Heroin |  |  |  |  |  |  |  |
| B | Methadone (not prescribed to you) |  |  |  |  |  |  |  |
| C | Other opiates/analgesics |  |  |  |  |  |  |  |
| D | Barbiturates |  |  |  |  |  |  |  |
| E | Sedatives/hypnotics/tranquilizers |  |  |  |  |  |  |  |
| F | Cocaine |  |  |  |  |  |  |  |
| G | Amphetamines |  |  |  |  |  |  |  |
| H | Cannabis |  |  |  |  |  |  |  |
| I | Hallucinogens |  |  |  |  |  |  |  |
| J | Inhalants |  |  |  |  |  |  |  |
| K | More than 1 substance per day (including alcohol) |  |  |  |  |  |  |  |

# Are you currently receiving any treatments for drug use?

[ ] Yes [ ] No [ ] Not applicable

# If Yes, receiving drug or alcohol treatment. What type(s) of treatment (choose all that apply)?

[ ] AA, NA, CA, or other self-help groups - NOT a group in a day drug treatment program

[ ] Individual or group counseling in a day drug treatment program

[ ] Pharmacologic medications (naltrexone, suboxone, methadone)

[ ] Other (specify)

# Were you required to receive treatment at prison/jail release? (as condition of parole or probation)

[ ] Yes [ ] No

# Do you currently see a substance use treatment provider?

[ ] Yes [ ] No

[ ] Don’t know /Not sure [ ] Not applicable

1. Were you mandated to attend any services at release other than substance use treatment ( for example anger management, job training etc)

[ ] Yes [ ] No

[ ] Don’t know /Not sure

*Now we will turn to some questions about your perceptions and reactions to life situations.*

# Section VI: Self-efficacy, Self-Management and Psychosocial Stress

1. General Self Efficacy Scale (GSE)

|  |  | Not at all true | Hardly true | Moderately true | Exactly true |
| --- | --- | --- | --- | --- | --- |
| A | I can always manage to solve difficult problems if I try hard enough. |  |  |  |  |
| B | If someone opposes me, I can find the means and ways to get what I want. |  |  |  |  |
| C | It is easy for me to stick to my aims and accomplish my goals. |  |  |  |  |
| D | I am confident that I could deal efficiently with unexpected events. |  |  |  |  |
| E | Thanks to my resourcefulness, I know how to handle unforeseen situations. |  |  |  |  |
| F | I can solve most problems if I invest the necessary effort. |  |  |  |  |
| G | I can remain calm when facing difficulties because I can rely on my coping abilities. |  |  |  |  |
| H | When I am confronted with a problem, I can usually find several solutions. |  |  |  |  |
| I | If I am in trouble, I can usually think of a solution. |  |  |  |  |
| J | I can usually handle whatever comes my way. |  |  |  |  |

| 1. Assume that the ladder below is way of picturing your life. The top of the ladder represents the best possible life for you. The bottom rung of the ladder represents the worst possible life for you.   Indicate where on the ladder you feel you personally stand right now by marking the circle  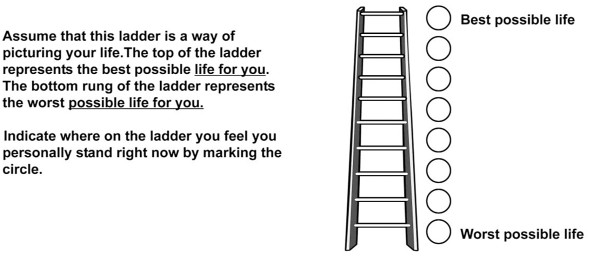   1. For each question below, please note how satisfied you are on a scale from 0 – 10 [Personal Wellbeing Index (PWI)] |
| --- |

|  |  | 0 | 1 | 2 | 3 | 4 | 5 | 6 | 7 | 8 | 9 | 10 |
| --- | --- | --- | --- | --- | --- | --- | --- | --- | --- | --- | --- | --- |
|  |  | Dissatisfied | |  |  |  | Neutral | |  |  | Satisfied | |
| A | Your standard of living? (the level of wealth, comfort and other necessities available to you) | □ | □ | □ | □ | □ | □ | □ | □ | □ | □ | □ |
| B | Your health? | □ | □ | □ | □ | □ | □ | □ | □ | □ | □ | □ |
| C | What you are achieving in life? | □ | □ | □ | □ | □ | □ | □ | □ | □ | □ | □ |
| D | Your personal relationships? | □ | □ | □ | □ | □ | □ | □ | □ | □ | □ | □ |
| E | How safe you feel? | □ | □ | □ | □ | □ | □ | □ | □ | □ | □ | □ |
| F | Feeling part of your community? | □ | □ | □ | □ | □ | □ | □ | □ | □ | □ | □ |
| G | Your future security | □ | □ | □ | □ | □ | □ | □ | □ | □ | □ | □ |
| H | Your spirituality or religion? | □ | □ | □ | □ | □ | □ | □ | □ | □ | □ | □ |

1. In your day-to-day life since release from prison/jail, how often do any of the following things happen to you? (*Everyday Discrimination Scale*).

|  |  | Never | Less than  1 time/week | 2 – 3 times /week | 4 – 5 times /week | More than 5 times/week | Almost  everyday |
| --- | --- | --- | --- | --- | --- | --- | --- |
|  |  |  |  |  |  |  |  |
| A | You are treated with less courtesy than other people are. |  |  |  |  |  |  |
| B | You are treated with less respect than other people are. |  |  |  |  |  |  |
| C | You receive poorer service than other people at restaurants or stores. |  |  |  |  |  |  |
| D | People act as if they think you are not smart. |  |  |  |  |  |  |
| E | People act as if they are afraid of you. |  |  |  |  |  |  |
| F | People act as if they think you are dishonest. |  |  |  |  |  |  |
| G | People act as if they’re better than you are. |  |  |  |  |  |  |
| H | You are called names or insulted. |  |  |  |  |  |  |
| I | You are threatened or harassed. |  |  |  |  |  |  |

***If the respondent answered “2-3 times” or more frequently to any question 87 above:***

1. What do you think is the main reason for these experiences? (Check more than one if volunteered)

[ ] Your Ancestry or National Origins [ ] Your Gender

[ ] Your Race [ ] Your Age

[ ] Your Religion [ ] Your Height

[ ] Your Weight [ ] Your shade of skin color

[ ] Some other Aspect of Your Physical Appearance [ ] Your Sexual Orientation

[ ] Your Education or Income Level [ ] A physical disability

[ ] History of incarceration

[ ] Other (SPECIFY)

# Please answer the following questions considering your experiences *since release from prison/jail* (Perceived Stress Scale (PSS)).

|  |  | Never | Almost never | Sometimes | Fairly Often | Very Often |
| --- | --- | --- | --- | --- | --- | --- |
| A | How often have you been upset because of something that happened unexpectedly? |  |  |  |  |  |
| B | How often have you felt that you were unable to control the important things in your life? |  |  |  |  |  |
| C | How often have you felt nervous and “stressed”? |  |  |  |  |  |
| D | How often have you felt confident about your ability to handle your personal problems? |  |  |  |  |  |
| E | How often have you felt that things were going your way? |  |  |  |  |  |
| F | How often have you found that you could not cope with all the things that you had to do? |  |  |  |  |  |
| G | How often have you been able to control irritations in your life? |  |  |  |  |  |
| H | How often have you felt that you were on top of things? |  |  |  |  |  |
| I | How often have you been angered because of things that were outside of your control? |  |  |  |  |  |
| J | How often have you felt difficulties were piling up so high that you could not overcome them? |  |  |  |  |  |

# Please answer the following questions considering your life time experiences. *(Cumulative Adversity Interview (CAI)*

|  |  | No | Yes | **If Yes,** did this occur while you were in prison/jail | | |
| --- | --- | --- | --- | --- | --- | --- |
|  |  |  |  | Yes | No | Don't know |
| A | Did you ever fail a grade in school? |  |  |  |  |  |
| B | Did your father or mother not have a job for a long time when they wanted to be working? |  |  |  |  |  |
| C | Were you ever sent away from home or kicked out of the house because you did something wrong? |  |  |  |  |  |
| D | Were you ever abandoned by one or both of your parents? |  |  |  |  |  |
| E | As a child, did you ever live in an orphanage, a foster home, a group home, or were a ward of the state? |  |  |  |  |  |
| F | Were you ever forced to live apart from one or both of your parents? |  |  |  |  |  |
| G | Did your parents ever divorce or separate? |  |  |  |  |  |
| H | Have you ever had a child who died at or near birth, or one that was taken away from you? |  |  |  |  |  |
| I | Have you ever discovered your spouse/boyfriend /girlfriend was unfaithful? |  |  |  |  |  |
| J | Did you ever lose your home because of a natural disaster? |  |  |  |  |  |
| K | Have you ever had a serious accident, injury or illness that was life threatening or caused long-term disability? |  |  |  |  |  |
| L | Did you ever have sexual intercourse when you did not want to because someone forced you or threatened to harm you if you did not? |  |  |  |  |  |
| M | Were you ever touched or made to touch someone else in a sexual way because they forced you in some way, or threatened to harm you if you did not? |  |  |  |  |  |
| N | Were you regularly physically abused by one of your parents, step-parents, grandparents or guardians? |  |  |  |  |  |
| O | Were you regularly emotionally abused by one of your caretakers? |  |  |  |  |  |
| P | Were you ever physically abused or injured by a spouse/boyfriend/girlfriend? |  |  |  |  |  |
| Q | Were you ever physically abused or injured by someone else you knew? |  |  |  |  |  |
| R | Have you ever been shot at with a gun or threatened with another weapon but not injured? |  |  |  |  |  |
| S | Have you ever been shot with a gun or badly injured with another weapon? |  |  |  |  |  |
| T | Have you ever been chased but not caught when you thought you could really get hurt? |  |  |  |  |  |
| U | Have you ever been physically assaulted or mugged? |  |  |  |  |  |
| V | Have you ever been in a car crash in which someone was killed or badly injured? |  |  |  |  |  |
| W | Have you ever witnessed a serious accident or disaster where someone else was hurt very badly or killed? |  |  |  |  |  |
| X | Did you witness your mother or another close female relative being regularly physically or emotionally abused? |  |  |  |  |  |
| Y | Have you seen someone chased but not caught or threatened with serious harm? |  |  |  |  |  |
| Z | Have you seen someone else get shot at or attacked with another weapon? |  |  |  |  |  |
| AA | Have you ever seen someone seriously injured by a gunshot or some other weapon? |  |  |  |  |  |
| AB | Have you ever actually seen someone get killed by being shot, stabbed, or beaten? |  |  |  |  |  |
| AC | Have you ever been told that someone you knew had been shot, but not killed? |  |  |  |  |  |
| AD | Have you ever been told that someone you knew had been killed with a gun or other weapon? |  |  |  |  |  |
| AE | Has anyone else you knew died suddenly or been seriously hurt? |  |  |  |  |  |
| AF | Have you ever been told that someone you knew killed him- or herself? |  |  |  |  |  |
| AG | Have you ever been told that someone you knew had been raped? |  |  |  |  |  |

1. Please indicate how often you feel the way described in each of the following statements *since release from prison/jail.*

|  |  | Never | Rarely | Sometimes | Usually | Always |
| --- | --- | --- | --- | --- | --- | --- |
| A | I feel left out |  |  |  |  |  |
| B | I feel that people barely know me |  |  |  |  |  |
| C | I feel isolated from others |  |  |  |  |  |
| D | I feel that people are around me but not with me |  |  |  |  |  |

# Section VII: Physical Activity

1. Since release from prison/jail, outside of work did you participate in any vigorous activities that increase your heart rate, or make you sweat doing them, or make you breathe hard such as running, gardening, or walking for exercise?

[ ] Yes

[ ] No

[ ] Don’t know/ Not sure **(If No, or Don’t know/not sure, SKIP to question 97)**

1. What type of physical activity or exercise did you spend the most time doing?

[ ]_running [ ]walking [ ]biking [ ]gardening [ ] other__________________

1. How many times per day or week did you take part in this activity

_____ times per day

_____ times per week

1. When you took part in this activity, for how many minutes or hours did you usually keep at it? ____ hours ____minutes [ ] Don’t know/Not sure
2. Since release how many times per day or week did you do physical activities or exercises to STRENGTHEN your muscles? *Do NOT count aerobic activities like walking, running, or bicycling. Count activities using your own body weight like yoga, sit-ups, or push-ups and those using weight machines, free weights, or elastic bands.*

_____ times per day

_____ times per week

[ ] Never

1. Neighborhood safety (*By your neighborhood we mean ALL the area within approximately half a mile of your home or that you could walk to in 10-15 minutes).*

***Please circle one answer per statement***

|  | Strongly disagree | Somewhat disagree | Somewhat agree | Strongly agree |
| --- | --- | --- | --- | --- |
| a) It is dangerous to leave a bicycle locked in my neighborhood |  |  |  |  |
| b) There are not enough safe places to cross busy streets in my neighborhood |  |  |  |  |
| c) Walking is dangerous because of the traffic in my neighborhood |  |  |  |  |
| d) Cycling is dangerous because of the traffic in my neighborhood |  |  |  |  |
| e) It is dangerous in my neighborhood during the day because of the level of crime |  |  |  |  |
| f) It is dangerous in my neighborhood during the night because of the level of crime |  |  |  |  |

# On the average, about how many hours per day do you watch television or other video programming?

______hours

# Section VIII: Diet Practices: Think about what you usually eat since release.

1. Which of the following statements best describes the nutritional quality of your diet?

 I really watch what I eat

 I am generally careful about what I eat

 It’s not all bad, but it could be better

 I rarely pay attention to nutrition

1. How often do you use the food label to make decisions about purchasing a food product?

 Always

 Most of the time

 Sometimes

 Rarely

 Never

 Not applicable

1. What are the factors of greatest interest to you that help you decide whether or not to purchase the food?**(CHECK ALL THAT APPLY)**

 Calories per serving

 Sodium

 Fat

 Sugar

 Fiber

 Cost

 Other

1. Thinking about how often you eat out, how many times in a week do you eat breakfast,

lunch, or dinner out in a place such as McDonald’s, Burger King, Wendy’s, Arby’s, Pizza Hut, or

Kentucky Fried Chicken, since release from jail/prison? ______ times per week

101a. How many of these meals are breakfast? _____

101b. How many of these meals are lunch? _______

101c. How many of these meals are dinner? _______

101d. How many were snacks? ______

1. Since release from prison/jail, how often did you eat the following:

|  | Never or < 1 per week | 1 -2 per week | 3 – 4 per week | 5 – 6 per week | 1 per day | 2 per day | 3 per day | 4 per day | 5+ per day |
| --- | --- | --- | --- | --- | --- | --- | --- | --- | --- |
| Foods cooked in fat (pan-fried, sautéed or deep fried) |  |  |  |  |  |  |  |  |  |
| A serving of vegetables? (don’t count salad, beans or potatoes) |  |  |  |  |  |  |  |  |  |
| A serving of fruit? (don’t count juice) |  |  |  |  |  |  |  |  |  |

# Section IX: Knowledge and Perceptions of Medical Conditions

1. Brief Illness Perception Questionnaire: For the following questions, please circle the number that best corresponds to your views.

|  |  | 0 | 1 | 2 | 3 | 4 | 5 | 6 | 7 | 8 | 9 | 10 |
| --- | --- | --- | --- | --- | --- | --- | --- | --- | --- | --- | --- | --- |
| A | How much does your illness affect your life? | No affect at all  □ | □ | □ | □ | □ | □ | □ | □ | □ | □ | Severely affects my life  □ |
| B | How long do you think your illness will continue? | A very short time  □ | □ | □ | □ | □ | □ | □ | □ | □ | □ | Forever  □ |
| C | How much control do you feel you have over your illness? | Absolutely no control  □ | □ | □ | □ | □ | □ | □ | □ | □ | □ | Extreme amount of control  □ |
| D | How much do you think your treatment can help your illness? | Not at all  □ | □ | □ | □ | □ | □ | □ | □ | □ | □ | Extremely helpful  □ |
| E | How much do you experience symptoms from your illness? | No symptoms at all  □ | □ | □ | □ | □ | □ | □ | □ | □ | □ | Many severe symptoms  □ |
| F | How concerned are you about your illness? | Not at all concerned  □ | □ | □ | □ | □ | □ | □ | □ | □ | □ | Extremely concerned  □ |
| G | How well do you feel you understand your illness? | Don’t understand at all  □ | □ | □ | □ | □ | □ | □ | □ | □ | □ | Understand very clearly  □ |
| H | How much does your illness affect you emotionally? (e.g. does it make you angry, scared, upset or depressed? | Not all affected  □ | □ | □ | □ | □ | □ | □ | □ | □ | □ | Extremely affected  □ |
| I | Please list in rank-order the three most important factors that you believe caused your illness. The most important causes for me: -   1. _______________________________________ 2. ______________________________________ 3. _______________________________________ | | | | | | | | | | | |

*I will now ask you about some common issues that individuals may face when they return home from prison/jail..*

1. Do you/Do you have/Are you:

|  | Issue | Yes | No | Don’t Know | N/A |
| --- | --- | --- | --- | --- | --- |
| A | Issues with debt collection or wage garnishment |  |  |  |  |
| B | Credit or financial reporting problems |  |  |  |  |
| C | Behind on utility payments/bills |  |  |  |  |
| D | Ongoing criminal fines or court fees |  |  |  |  |
| E | Trying to restore a license (e.g. driver’s license, commercial driver’s license) |  |  |  |  |
| F | Need to obtain/preserve/increase disability benefits |  |  |  |  |
| G | Need to obtain/preserve/increase worker’s comp or unemployment benefits |  |  |  |  |
| H | Having trouble finding a job due to your criminal record |  |  |  |  |
| I | Getting paid less than you expect or late |  |  |  |  |
| J | Child support/alimony issues |  |  |  |  |
| K | Child visitation or custody problems |  |  |  |  |
| L | Need a protective order (e.g. restraining order) |  |  |  |  |
| M | Having difficulty with your landlord or roommates |  |  |  |  |
| N | Having trouble paying rent or at risk for eviction from current living situation or foreclosure on home |  |  |  |  |
| O | Unsafe living conditions (e.g. mold, violence, lack of heat, overcrowding) |  |  |  |  |
| P | Need to obtain/preserve/increase certain public benefits (e.g. food stamps, welfare, SSD/SSI) |  |  |  |  |
| Q | Need help with medical bills |  |  |  |  |
| R | Need to obtain/preserve/increase health insurance benefits (e.g. Medicare/Medicaid) |  |  |  |  |
