# Supplementary material for: A prospective cohort study examining exposure to incarceration and cardiovascular disease (Justice-Involved Individuals Cardiovascular Disease Epidemiology – JUSTICE study): a protocol paper
Source: BMC Public Health. 2022 Feb 16;22:331. doi: 10.1186/s12889-022-12688-x (PMC8848673; doi:10.1186/s12889-022-12688-x)
Supplement: Supplementary file 2 — Additional file 2. [file 12889_2022_12688_MOESM2_ESM.docx]

# JUSTICE study Follow up survey

Participant ID number:______________

Initials of Interviewer: ___________

Follow up time point: Six months ___ 12 months ___

Date (mm/dd/yyyy) ____________________

|  |
| --- |
|  |

# Is participant currently incarcerated? (Base: All participants)

# [ ] Yes (Skip to Q9)

# [ ] No

# Where do you live now? (If response is unclear, prompt to select category) (Base: Q1=No)

[ ] Homeless living on the street, park, bus station, etc.(living outdoors)

[ ] Homeless in a shelter

[ ] Drug treatment facility

[ ] Other residential facility or institution (e.g. mental health facility, halfway house)

[ ] Staying with family/friends

[ ] Rent an apartment/house

[ ] Own my home

[ ] Other (please specify) : ______________

1. Do you have health insurance? (Base :Q1=No)

[ ]Yes  [ ] No [ ] Don’t know/ Not sure

1. What kind of health insurance? (select all that apply) (base: Q3=yes)

[ ] Medicaid

[ ] Medicare

[ ] Employer-provided insurance

[ ] VA Insurance

[ ] Other (please specify): ____________________

1. Are you currently receiving food stamps (SNAP)? (Base: q1=No)

[ ] Yes [ ] No [ ] Don’t know/Not sure

5a. What are the reasons you are not getting foods stamps? (base: q5=No)

[ ] Have not applied for food stamps [ ] Pending application

[ ] Criminal record (e.g. drug felony conviction) [ ] Don’t know

[ ] Other __________________________

1. Have you gone a whole day without food in the last 6 months because you did not have enough money to feed yourself? (Base: Q1=No)

[ ] Yes [ ] No **(SKIP to Q7)**

6a.**What** is the reason you went for a whole day without food? (base: q5=yes and q6=yes)

[ ] Not enough to feed for a month [ ] Exchanged food stamps for alcohol/tobacco/drugs

[ ] Exchanged food stamps for other needs [ ] Other reason

# Last week, did you do any work for pay or profit? ( *Unpaid work in a family business is considered work.)* (Base: Not incarcerated (Q1=No))

[ ] Yes [ ] No

[ ] Prefer not to answer

# What is your average monthly income *(include disability, food stamps, child support, workers comp, unemployment, any pensions, and money from family/friends)*? (Base: Q1=No)

[ ] No income [ ] $1-$99

[ ] $100-$499 [ ] $500-$999

[ ] $1000-$1999 [ ] $2000-$4999

[ ] $5000 or more [ ] Prefer not to say

# In general, would you say your health is (Base: all participants)

# [ ] Excellent

# [ ] Very Good

# [ ] Good

# [ ] Fair

# [ ] Poor

1. Over the last 2 weeks, how often have you been bothered by any of the following problems? (base: all participants)

*[If patient responds not at all and/or several days to both questions a and b, skip to Q.56)*

| Over the last 2 weeks, how often have you been bothered by any of the following problems? | Not at all | Several days | More than half the days | Nearly everyday |
| --- | --- | --- | --- | --- |
| a. Little interest or pleasure in doing things |  |  |  |  |
| b. Feeling down, depressed, or hopeless |  |  |  |  |
| c. Trouble falling or staying asleep, or sleeping too much |  |  |  |  |
| d. Feeling tired or having little energy |  |  |  |  |
| e. Poor appetite or overeating |  |  |  |  |
| f. Feeling bad about yourself - or that you are a failure or have let yourself or your family down |  |  |  |  |
| g. Trouble concentrating on things, such as reading the newspaper or watching television |  |  |  |  |
| h. Moving or speaking so slowly that other people might have noticed? Or the opposite - being so fidgety or restless that you have been moving around a lot more than usual |  |  |  |  |
| i. Thoughts that you would be better off dead or of hurting yourself in some way |  |  |  |  |

# Have you been to an emergency department in the past 3 months? (base: all participants)

[ ] Yes [ ] No **(skip to Q13)**

# 11a: How many times ? (base: q11=yes)

1. Have you been hospitalized (24 hours/admitted overnight) in the past 3 months? (base: all participants)

[ ] Yes [ ] No

1. In the past 6 months, have you ever been arrested, booked, or charged for breaking a law? (base: q1=no)

[ ] Yes [ ] No

1. In the past 6 months, have you ever been convicted of or pled guilty to any charges other than a minor traffic violation? (base: q1=no)

[ ] Yes [ ] No

1. In the last 6 months, have you spent any time in a jail, prison? (base: q1=no)

[ ] Yes [ ] No

1. Do you have a primary care doctor (A doctor you go to regularly if you need check-up or are ill)? (base: all )

[ ] Yes [ ] No [ ] Don’t know /Not sure

1. In the past 6 months, have you seen a primary care doctor/provider? (base: all )

[ ] Yes [ ] No **(skip to Q16)**

14a. In the past 6 months, how many times have you seen a doctor/provider? (base: q17=yes)_________

14b: How many of these were during incarceration? (base: q1=yes or q15=yes)

1. W*hen* I received care for my chronic conditions, I was:  *(PACIC)*  (*base: q17=yes)*

|  |  | None of the time | A little of the time | Some of the time | Most of the time | Always |
| --- | --- | --- | --- | --- | --- | --- |
| A | Asked for my ideas when we made a treatment plan |  |  |  |  |  |
| B | Given choices about treatment to think about. |  |  |  |  |  |
| C | Asked to talk about any problems with my medicines or their effects. |  |  |  |  |  |
| D | Given a written list of things I should do to improve my health. |  |  |  |  |  |
| E | Satisfied that my care was well organized. |  |  |  |  |  |
| F | Shown how what I did to take care of myself influenced my condition. |  |  |  |  |  |
| G | Asked to talk about my goals in caring for my condition. |  |  |  |  |  |
| H | Helped to set specific goals to improve my eating or exercise. |  |  |  |  |  |
| I | Given a copy of my treatment plan |  |  |  |  |  |
| J | Encouraged to go to a specific group or class to help me cope with my chronic condition. |  |  |  |  |  |
| K | Asked questions, either directly or on a survey, about my health habits. |  |  |  |  |  |
| L | Sure that my doctor or nurse thought about my values, beliefs, and traditions when they recommended treatments to me. |  |  |  |  |  |
| M | Helped to make a treatment plan that I could carry out in my daily life. |  |  |  |  |  |
| N | Helped to plan ahead so I could take care of my condition even in hard times. |  |  |  |  |  |
| O | Asked how my chronic condition affects my life. |  |  |  |  |  |
| P | Contacted after a visit to see how things were going. |  |  |  |  |  |
| Q | Encouraged to attend programs in the community that could help me. |  |  |  |  |  |
| R | Referred to a dietitian, health educator, or counselor. |  |  |  |  |  |
| S | Told how my visits with other types of doctors, like an eye doctor or other specialist, helped my treatment. |  |  |  |  |  |
| T | Asked how my visits with other doctors were going. |  |  |  |  |  |

1. Are you currently taking any medications that you were told to take regularly by a healthcare provider? (base: q17=yes)

[ ] Yes [ ] No [ ] Refused

1. Abbreviated (4 item) Morisky scale (base: q19=yes)

|  | Yes | No |
| --- | --- | --- |
| a. Do you ever forget to take your medications? |  |  |
| b. Are you careless at times about taking your medications? |  |  |
| c. When you feel better do you stop taking your medications? |  |  |
| d. Sometimes if you feel worse when you take your medication, do you stop taking it? |  |  |

#

***Now we will turn to a few questions about smoking, alcohol use, illicit drug use, and your activities in the past six months.***

# Tobacco Use

# Do you currently smoke cigarettes? (base: all)

[ ] Yes [ ] No **(skip to 20)**

# On average, about how many cigarettes a day do you now smoke? (base: q21=yes) cigarettes

# Alcohol Use

# Please answer the following questions about your alcohol use in the past 6 months (AUDIT-C) (Base: all)

|  | Question | Response Options | | | | |
| --- | --- | --- | --- | --- | --- | --- |
| A | How often do you have a drink containing alcohol? | Never  □  \\ | rarely (<1/week) (monthly or less)  □ | about once a week (2-4x/mo)  □ | 2 to 3 times a  Week  □ | 4 or more times a week  □ |
| B | How many standard drinks containing alcohol do you have on a typical day? | 1 or 2  □ | 3 or 4  □ | 5 or 6  □ | 7 to 9  □ | 10 or more  □ |
| C | How often do you have six or more drinks on one occasion? | Never  □ | less than monthly  □ | Monthly  □ | Weekly  □ | daily or almost daily  □ |

# Illicit Drug Use

1. For each of the following drugs, indicated use in the past 6 months and the usual route. (Base: all)

|  |  |  | Note the usual or most recent route. *For more than one route, choose the most severe. The routes are listed from least severe (left) to most severe (right)* | | | | |
| --- | --- | --- | --- | --- | --- | --- | --- |
|  | Name of Drug | Used in past 6 months (days) | Oral | Nasal | Smoking | Non-IV injection | IV |
| A | Heroin |  |  |  |  |  |  |
| B | Methadone (not prescribed to you) |  |  |  |  |  |  |
| C | Other opiates/analgesics |  |  |  |  |  |  |
| D | Barbiturates |  |  |  |  |  |  |
| E | Sedatives/hypnotics/tranquilizers |  |  |  |  |  |  |
| F | Cocaine |  |  |  |  |  |  |
| G | Amphetamines |  |  |  |  |  |  |
| H | Cannabis |  |  |  |  |  |  |
| I | Hallucinogens |  |  |  |  |  |  |
| J | Inhalants |  |  |  |  |  |  |
| K | More than 1 substance per day (including alcohol) |  |  |  |  |  |  |

# Are you currently receiving any treatments (medical or counseling) for drug use? (base: all)

[ ] Yes [ ] No [ ] Not applicable

# What type(s) of treatment (choose all that apply)? (base: q25=yes)

[ ] AA, NA, CA, or other self-help groups - NOT a group in a day drug treatment program

[ ] Individual or group counseling in a day drug treatment program

[ ] Pharmacologic medications (naltrexone, suboxone, methadone)

[ ] Other (specify)

*Now we will turn to some questions about your perceptions and reactions to life situations.*

1. General Self Efficacy Scale (GSE) (base: all)

|  |  | Not at all true | Hardly true | Moderately true | Exactly true |
| --- | --- | --- | --- | --- | --- |
| A | I can always manage to solve difficult problems if I try hard enough. |  |  |  |  |
| B | If someone opposes me, I can find the means and ways to get what I want. |  |  |  |  |
| C | It is easy for me to stick to my aims and accomplish my goals. |  |  |  |  |
| D | I am confident that I could deal efficiently with unexpected events. |  |  |  |  |
| E | Thanks to my resourcefulness, I know how to handle unforeseen situations. |  |  |  |  |
| F | I can solve most problems if I invest the necessary effort. |  |  |  |  |
| G | I can remain calm when facing difficulties because I can rely on my coping abilities. |  |  |  |  |
| H | When I am confronted with a problem, I can usually find several solutions. |  |  |  |  |
| I | If I am in trouble, I can usually think of a solution. |  |  |  |  |
| J | I can usually handle whatever comes my way. |  |  |  |  |

| 1. For each question below, please note how satisfied you are on a scale from 0 – 10 (PWI) (base: all) |
| --- |

|  |  | 0 | 1 | 2 | 3 | 4 | 5 | 6 | 7 | 8 | 9 | 10 |
| --- | --- | --- | --- | --- | --- | --- | --- | --- | --- | --- | --- | --- |
|  |  | Dissatisfied | |  |  |  | Neutral | |  |  | Satisfied | |
| A | Your standard of living? (the level of wealth, comfort and other necessities available to you) | □ | □ | □ | □ | □ | □ | □ | □ | □ | □ | □ |
| B | Your health? | □ | □ | □ | □ | □ | □ | □ | □ | □ | □ | □ |
| C | What you are achieving in life? | □ | □ | □ | □ | □ | □ | □ | □ | □ | □ | □ |
| D | Your personal relationships? | □ | □ | □ | □ | □ | □ | □ | □ | □ | □ | □ |
| E | How safe you feel? | □ | □ | □ | □ | □ | □ | □ | □ | □ | □ | □ |
| F | Feeling part of your community? | □ | □ | □ | □ | □ | □ | □ | □ | □ | □ | □ |
| G | Your future security | □ | □ | □ | □ | □ | □ | □ | □ | □ | □ | □ |
| H | Your spirituality or religion? | □ | □ | □ | □ | □ | □ | □ | □ | □ | □ | □ |

1. In your day-to-day life in the past 6 months, how often do any of the following things happen to you? (*Everyday Discrimination Scale*). **(Base: all )**

|  |  | Never | Less than  1 time/week | 2 – 3 times /week | 4 – 5 times /week | More than 5 times/week | Almost  everyday |
| --- | --- | --- | --- | --- | --- | --- | --- |
|  |  |  |  |  |  |  |  |
| A | You are treated with less courtesy than other people are. |  |  |  |  |  |  |
| B | You are treated with less respect than other people are. |  |  |  |  |  |  |
| C | You receive poorer service than other people at restaurants or stores. |  |  |  |  |  |  |
| D | People act as if they think you are not smart. |  |  |  |  |  |  |
| E | People act as if they are afraid of you. |  |  |  |  |  |  |
| F | People act as if they think you are dishonest. |  |  |  |  |  |  |
| G | People act as if they’re better than you are. |  |  |  |  |  |  |
| H | You are called names or insulted. |  |  |  |  |  |  |
| I | You are threatened or harassed. |  |  |  |  |  |  |

1. What do you think is the main reason for these experiences? (Check all that apply) (base: q29=2-3/wk)

[ ] Your Gender

[ ] Your Race

[ ] Your Age

[ ] Your shade of skin color

[ ] Your Sexual Orientation

[ ] History of incarceration

[ ] Other (SPECIFY)

# Please answer the following questions considering your experiences in the past 6 months (PSS) (base: all).

|  |  | Never | Almost never | Sometimes | Fairly Often | Very Often |
| --- | --- | --- | --- | --- | --- | --- |
| A | How often have you been upset because of something that happened unexpectedly? |  |  |  |  |  |
| B | How often have you felt that you were unable to control the important things in your life? |  |  |  |  |  |
| C | How often have you felt nervous and “stressed”? |  |  |  |  |  |
| D | How often have you felt confident about your ability to handle your personal problems? |  |  |  |  |  |
| E | How often have you felt that things were going your way? |  |  |  |  |  |
| F | How often have you found that you could not cope with all the things that you had to do? |  |  |  |  |  |
| G | How often have you been able to control irritations in your life? |  |  |  |  |  |
| H | How often have you felt that you were on top of things? |  |  |  |  |  |
| I | How often have you been angered because of things that were outside of your control? |  |  |  |  |  |
| J | How often have you felt difficulties were piling up so high that you could not overcome them? |  |  |  |  |  |

1. Please indicate how often you feel the way described in each of the following statements *in the past 6 months.* (base: all)

|  |  | Never | Rarely | Sometimes | Usually | Always |
| --- | --- | --- | --- | --- | --- | --- |
| A | I feel left out |  |  |  |  |  |
| B | I feel that people barely know me |  |  |  |  |  |
| C | I feel isolated from others |  |  |  |  |  |
| D | I feel that people are around me but not with me |  |  |  |  |  |

1. In the past six months, outside of work did you participate in any vigorous activities that increase your heart rate, or make you sweat doing them, or make you breathe hard such as running, gardening, or walking for exercise? (base: all)

[ ] Yes

[ ] No

[ ] Don’t know/ Not sure

1. What type of physical activity or exercise did you spend the most time doing? (base: q33=yes)

[ ]_running [ ]walking [ ]biking [ ]gardening [ ] other__________________

1. How many times per day or week did you take part in this activity (base: q33=yes)

_____ times per day

_____ times per week

1. When you took part in this activity, for how many minutes or hours did you usually keep at it? (base: q33=yes) ____ hours ____minutes [ ] Don’t know/Not sure
2. In the past six months how many times per day or week did you do physical activities or exercises to STRENGTHEN your muscles *(like yoga, sit-ups, push-ups and activities using weight machines, free weights, or elastic bands)? (base: all)*

_____ times per day

_____ times per week

[ ] Never

1. Neighborhood safety (*By your neighborhood we mean ALL the area within approximately half a mile of your home or that you could walk to in 10-15 minutes).* **(Base: q1=No)**

***Please circle one answer per statement***

|  | Strongly disagree | Somewhat disagree | Somewhat agree | Strongly agree |
| --- | --- | --- | --- | --- |
| a) It is dangerous to leave a bicycle locked in my neighborhood |  |  |  |  |
| b) There are not enough safe places to cross busy streets in my neighborhood |  |  |  |  |
| c) Walking is dangerous because of the traffic in my neighborhood |  |  |  |  |
| d) Cycling is dangerous because of the traffic in my neighborhood |  |  |  |  |
| e) It is dangerous in my neighborhood during the day because of the level of crime |  |  |  |  |
| f) It is dangerous in my neighborhood during the night because of the level of crime |  |  |  |  |

# On the average, about how many hours per day do you watch television or other video programming? (base : all) _________ hours

# Diet Practices: Think about what you usually eat in the past six months.

1. Which of the following statements best describes the nutritional quality of your diet?

[ ] I really watch what I eat

[ ] I am generally careful about what I eat

[ ] It’s not all bad, but it could be better

[ ] I rarely pay attention to nutrition

1. How often do you use the food label to make decisions about purchasing a food product? (base: all)

[ ] Always

[ ] Most of the time

[ ] Sometimes

[ ] Rarely

[ ] Never

[ ] Not applicable

1. What are the factors of greatest interest to you that help you decide whether or not to purchase the food?**(CHECK ALL THAT APPLY)** (base: all)

[ ] Calories per serving

[ ] Sodium

[ ] Fat

[ ] Sugar

[ ] Fiber

[ ] Cost

[ ] Other

1. Thinking about how often you eat out, how many times in a week do you eat breakfast,

lunch, or dinner out in a place such as McDonald’s, Burger King, Wendy’s, Arby’s, Pizza Hut, or

Kentucky Fried Chicken, in the past six months? ______ times per week (base: q1=no)

a. How many of these meals are breakfast? _____

b. How many of these meals are lunch? _______

c. How many of these meals are dinner? _______

d. How many were snacks? ______

1. In the past six months, how often did you eat the following: (Base: ALL)

|  | Never or < 1 per week | 1 -2 per week | 3 – 4 per week | 5 – 6 per week | 1 per day | 2 per day | 3 per day | 4 per day | 5+ per day |
| --- | --- | --- | --- | --- | --- | --- | --- | --- | --- |
| Foods cooked in fat (pan-fried, sautéed or deep fried) |  |  |  |  |  |  |  |  |  |
| A serving of vegetables? (don’t count salad, beans or potatoes) |  |  |  |  |  |  |  |  |  |
| A serving of fruit? (don’t count juice) |  |  |  |  |  |  |  |  |  |

1. **Brief Illness Perception Questionnaire:**  For the following questions, please circle the number that best corresponds to your views. (Base: ALL)

|  |  | 0 | 1 | 2 | 3 | 4 | 5 | 6 | 7 | 8 | 9 | 10 |
| --- | --- | --- | --- | --- | --- | --- | --- | --- | --- | --- | --- | --- |
| A | How much does your illness affect your life? | No affect at all  □ | □ | □ | □ | □ | □ | □ | □ | □ | □ | Severely affects my life  □ |
| B | How long do you think your illness will continue? | A very short time  □ | □ | □ | □ | □ | □ | □ | □ | □ | □ | Forever  □ |
| C | How much control do you feel you have over your illness? | Absolutely no control  □ | □ | □ | □ | □ | □ | □ | □ | □ | □ | Extreme amount of control  □ |
| D | How much do you think your treatment can help your illness? | Not at all  □ | □ | □ | □ | □ | □ | □ | □ | □ | □ | Extremely helpful  □ |
| E | How much do you experience symptoms from your illness? | No symptoms at all  □ | □ | □ | □ | □ | □ | □ | □ | □ | □ | Many severe symptoms  □ |
| F | How concerned are you about your illness? | Not at all concerned  □ | □ | □ | □ | □ | □ | □ | □ | □ | □ | Extremely concerned  □ |
| G | How well do you feel you understand your illness? | Don’t understand at all  □ | □ | □ | □ | □ | □ | □ | □ | □ | □ | Understand very clearly  □ |
| H | How much does your illness affect you emotionally? (e.g. does it make you angry, scared, upset or depressed? | Not all affected  □ | □ | □ | □ | □ | □ | □ | □ | □ | □ | Extremely affected  □ |
| I | Please list in rank-order the three most important factors that you believe caused your illness. The most important causes for me: -   1. _______________________________________ 2. ______________________________________ 3. _______________________________________ | | | | | | | | | | | |

*I will now ask you about some common issues that individuals may face when they return home from prison/jail.*

1. Do you/Do you have/Are you: (Base: q1=no )

|  | Issue | Yes | No | Don’t Know | N/A |
| --- | --- | --- | --- | --- | --- |
| A | Issues with debt collection or wage garnishment |  |  |  |  |
| B | Credit or financial reporting problems |  |  |  |  |
| C | Behind on utility payments/bills |  |  |  |  |
| D | Ongoing criminal fines or court fees |  |  |  |  |
| E | Trying to restore a license (e.g. driver’s license, commercial driver’s license) |  |  |  |  |
| F | Need to obtain/preserve/increase disability benefits |  |  |  |  |
| G | Need to obtain/preserve/increase worker’s comp or unemployment benefits |  |  |  |  |
| H | Having trouble finding a job due to your criminal record |  |  |  |  |
| I | Getting paid less than you expect or late |  |  |  |  |
| J | Child support/alimony issues |  |  |  |  |
| K | Child visitation or custody problems |  |  |  |  |
| L | Need a protective order (e.g. restraining order) |  |  |  |  |
| M | Having difficulty with your landlord or roommates |  |  |  |  |
| N | Having trouble paying rent or at risk for eviction from current living situation or foreclosure on home |  |  |  |  |
| O | Unsafe living conditions (e.g. mold, violence, lack of heat, overcrowding) |  |  |  |  |
| P | Need to obtain/preserve/increase certain public benefits (e.g. food stamps, welfare, SSD/SSI) |  |  |  |  |
| Q | Need help with medical bills |  |  |  |  |
| R | Need to obtain/preserve/increase health insurance benefits (e.g. Medicare/Medicaid) |  |  |  |  |

# Now we will ask a few questions about your most recent incarceration.

1. Where did you serve your most recent incarceration? **(base: q15=yes)** [ ] Prison [ ] Jail
2. When did you start your last prison/jail term [the one that just ended]? **(base: q15=yes)** ____________ (mm/dd/yyyy)
3. When were you released from your last prison/jail term? **(base: q15=yes)**_____________ (mm/dd/yyyy)

# Are you currently on? (select all that apply) (base: q15=yes)

[ ] Parole [ ] Probation [ ] Supervised Release

# If yes, when does the parole/probation/supervised release end? [If you are on both parole and probation, choose the furthest out date.] _____________ (mm/dd/yyyy)

1. During your last prison/jail term [the one that just ended], were you ever placed in restricted status (e.g. solitary, the hold, Seg, AdSeg, the SHU)? **(base: q15=yes)**

[ ] Yes [ ] No (skip to Q31) [ ] Don’t know (skip to Q31)

# What is the total time that you spent on restricted status during your last prison/jail term? (base: q51=yes)

[ ] 1 week or less [ ] 1-4 weeks

[ ] 1 to 3 months [ ] 3 to 6 months

[ ] 6 months-1 year

# Did you see a health care provider for a chronic health condition during your most recent prison/jail term? (base: q15=yes)

[ ] Yes [ ] No (skip to Q39)

# Were you ever treated unfairly by healthcare providers (doctors, nurses, etc.)

# during your last prison/jail stay? (base: q53=yes)

[ ] Yes [ ] No [ ] Not applicable

# Are there any medications that you were told to take regularly by the healthcare provider working with you during the last prison/jail term? (base: q53=yes)

[ ] Yes [ ] No (skip to Q39) [ ] Refused (skip to Q39)

# Were you allowed to keep on you any of the medications that you were told to take regularly by the healthcare provider during the last prison/jail term? (base: q53=yes)

[ ] Yes [ ] No

# Were you released from prison/jail with a medications voucher? (base: q53=yes)

[ ] Yes [ ] No (skip to Q39) [ ] Not sure(skip to Q39)

1. Approximately how many weeks of medication did you receive? _______ weeks (base: q57=yes)
2. Did you receive any education about caring for your disease condition [hypertension, diabetes, hyperlipidemia, obesity] during your most recent incarceration? (base: q15=yes)

[ ] Yes [ ] No [ ] Don’t know

1. Were you allowed exercise time during your most recent incarceration? **(base: q15=yes)**

[ ] Yes [ ] No – skip to question 47 [ ] Prefer not answer

1. How much time on average per week, were you allowed to exercise? (base: q60=yes)______ Hours ____Min
2. On average, how much time per week did you spend doing exercises that increase your heart rate, or make you sweat doing them, or make you breathe hard such as running, or playing basketball? (base: q60=yes) ________ Hours _______Min
3. On average, how much time per week did you spend doing exercises that build or strengthen your muscles such as pushups or weight lifting? (base: q60=yes) ________ Hours _______Min
